# Comparative elucidation of bioactive and volatile components in dry mature jujube fruit (*Ziziphus jujuba* Mill.) subjected to different drying methods

**DOI:** 10.1016/j.fochx.2022.100311

**Published:** 2022-04-18

**Authors:** Yuxing Liu, Yaxuan Liao, Minrui Guo, Weida Zhang, Yueying Sang, Hai Wang, Shaobo Cheng, Guogang Chen

**Affiliations:** a School of Food Science and Technology, Shihezi University, Shihezi 832000, China; b Academy of Agricultural Planning and Engineering, Beijing 100020, China

**Keywords:** Drying method, Convection drying, Biological activity, Volatile components, Fatty acid, Amino acid

## Abstract

This study investigated the effects of convective drying(CD) and freeze drying(FD) on bioactive and volatile components in jujube. No significant difference in total phenolic, total flavonoids and antioxidant capacity among CD60, CD70, CD80 and FD samples (*P* > 0.05). LC-MS/MS analysis showed that this trend mainly originated from the dynamic equilibrium relationships between caffeic acid, chlorogenic acid, *p*-hydroxybenzoic acid, rutin, epicatechin, and quercetin. HS-SPME-GC–MS identified 31 volatile organic compounds (VOCs) comprising more than 80% aldehydes and acids. Principal component analysis distinguished the VOC characteristics of samples subjected to different drying methods. Six VOCs had an odor activity value (OAV) >1, most of which were fatty acid oxidation or Maillard reaction products. Combined with the precursor components, these reactions were speculated to be the major VOC-producing pathways in dried jujube. Considering the bioactive components and flavor retention, CD at 60 °C was an effective drying method with potential to replace FD.

## Highlights

• Convective drying(CD) increased the concentrations of rutin, epicatechin and quercetin in jujube, and the total phenolic content was not significantly different with freeze-drying(FD).
• Six volatile components with OVA >1 were identified in dried jujube, and their concentration changes were related to precursor substances.
• The volatile components expressing “Fruity” aromas increased after CD, and the expression of fruity aromas at CD60 even exceeded that of FD samples.
• CD60 is an efficient drying method with potential to replace FD in terms of bioactivity and aroma.

## 1 Introduction

Jujube fruit (*Ziziphus jujuba* Mill.) is among the most consumed fruits in the Rhamnaceae family owing to its positive health effects and characteristic flavor. Further research found bioactive components with high antioxidant capacity, such as polysaccharides, polyphenolics, ascorbic acid and carotene, in jujube (Xie et al., 2017). These bioactive components have potential medicinal value, and can protect the body from oxidative stress-mediated toxicity and injury. According to reported statistics, China produced 8.5 million tons of jujube fruit in 2018, accounting for more than 90% of global production. Xinjiang province accounts for 49% of jujube fruit output in China (Pan et al., 2022). However, the postharvest stability of jujube fruit is poor and the shelf life is short, making long-term supply impossible (Song et al., 2020). To overcome this limitation, drying is a necessary processing method to realize the annual supply of processed raw materials, and to retain flavor and nutrition (Chumroenphat et al., 2021).

Drying, as a crucial postharvest step, is the oldest and most effective preservation technologies in the food industry (Zia & Alibas, 2021). It can inhibit enzymes and microbes, while maintaining the beneficial properties of the plant products (Alkaltham et al., 2021). Nowadays, a series of drying methods have been successfully applied to dry fresh jujube, such as hot air drying, microwave drying, vacuum freeze-drying, and infrared drying (Pu et al., 2018, Song et al., 2020). Unfortunately, the known methods are not suitable for industrialized food production owing to their high cost and difficult operation. As the largest jujube producing area, Xinjiang province in northwest China has developed a unique drying method that combines natural drying with convective drying (CD) for long-term planting according to the local climate characteristics. This method requires drying the raw jujube fruit on the tree until it falls off naturally. Previously, Fu et al. (2021) reported this process as the “dry mature stage of jujube”. Subsequently, the dried jujube is collected to further dehydrate with convective drying, which effectively improves the fruit flavor and texture (Liu et al., 2021c). However, little research has focused on the convective drying process, and the relationship between the convective drying parameters (such as temperature, time, and drying speed) and heat-sensitive components remains unknown.

Convective drying involves transferring heat from dry air to wet materials, which draws internal moisture to the fruit surface for evaporation (Ma et al., 2021). Generally, heat-sensitive components, such as bioactive components and volatile components, are irreversibly degraded or oxidized during the long, high-temperature dehydration process (Tan et al., 2021). During the drying process of jujube, Bao et al., 2021, Chen et al., 2015 found that phenolic compounds, including gallic acid, p-hydroxybenzoic acid, vanillic acid, p-coumaric acid, ferulic acid and rutin, were sensitive to drying methods and technological parameters. Meanwhile, the drying process will also change the proportion of volatile organic compounds (VOCs) with Fruit and Green aroma, such as (E)-2- hexenal, hexanal, (Z)-2- heptanal, benzaldehyde and (E)-2-nonenal (Chen et al., 2018, Song et al., 2020). Presently, most research indicates that freeze-drying (FD) is the best method for retaining bioactivity and VOCs. However, FD has the disadvantage of being inefficient and expensive. Therefore, a drying method is needed for jujube fruit that has a controllable cost and effective retention of fruit quality. Unfortunately, little was previously known about the drying characteristics of the dry mature stage of jujube fruit. Furthermore, to our knowledge, few studies have focused on the effects of CD and FD on the biological activity and VOCs of jujube fruit.

The development of a fast, convenient, and energy-efficient drying method is key to realizing the economic value of jujube fruit. According to the principles of economic efficiency and product quality, we believe that CD is still an effective process for the further dehydration of jujube fruit at the dry mature stage. To select the most suitable dehydration parameters for jujube fruit, this study investigated the effects of FD and CD parameters on the bioactive components and VOCs of jujube. This study aimed to preserve the bioactivity and flavor of jujube fruit and provide useful information on drying that can be applied on a large scale in industry.

## 2 Materials and methods

Jujube fruit (*Ziziphus jujuba* Mill. cv. Junzao) was harvested in December 2020 in Hotan, Xinjiang province (latitude, 79°18′22″; longitude, 37°13′9″). The local climate is temperate continental desert with a high temperature and dry climate, and the annual average rainfall is <50 mm. Approximately 10 kg of dry mature jujube fruit (wet base moisture content, 21%) was collected in foam boxes covered with ice. The samples were sent to the laboratory at Xinjiang Shihezi University within one day. The screened samples were cleaned with double-distilled water (ddH_2_O), and stored in a dry and odorless cold storage at 4 °C until drying and analysis were complete.

Caffeic acid, ferulic acid, phloroglucinol, cinnamic acid, chlorogenic acid, *p*-hydroxybenzoic acid, *p*-coumaric acid, vanillic acid, gallic acid, catechin, protocatechuic acid, epicatechin, rutin, quercetin, and procyanidins B1 and B2 were obtained from Anpel Co. (Shanghai, China). A total of 37 fatty acid mixed standards, 17 amino acid mixed standards, asparagine, glutamine, citrulline, valine, tryptophan, and 21 hydroxyproline and sarcosine standards were obtained from Sigma Aldrich (St. Louis., Mo, USA).

### 2.2 Drying conditions

FD samples were dried using a Scientz-10ND vacuum freeze-drying system (Scientz Co., Ningbo, China). The samples were pre-frozen with liquid nitrogen, evenly tiled at 50 g/dm^3^, and vacuum freeze-dried at −45 °C and 20 Pa.

CD samples were dried by a DGG-9053a convection drying oven (Sinxin Co., Shanghai, China). The samples were evenly tiled at 50 g/dm^3^ and convective-dried at 40–80 °C (denoted as CD40–80). According to parameters studied previously, drying experiments were conducted for 1440, 740, 300, 180, and 140 min (Liu et al., 2021c). The end point was 15% of the moisture content of the wet material.

### 2.3 Bioactive components

#### 2.3.1 Total phenolics, total flavonoids, and ascorbic acid

The total phenolics concentration (TPC) and total flavonoid concentration (TFC) were determined according to the method of Chumroenphat et al. (2021). The sample (5 g) was placed in methanol–water (80:20, v/v), extracted by ultrasonication (10 °C, 500 W, 30 min), and centrifuged at 8000 rpm for 10 min. The resulting residue was extracted repeatedly, and the volume was made up to 50 mL to obtain the extraction solution.

TPC determination was performed according to the Folin–Ciocalteu reaction. Briefly, to the extract (0.1 mL) was added Folin–Ciocalteu reagent (0.5 mL) and 10% Na_2_CO_3_ (1.5 mL). The TPC of the sample was quantified by constructing standard curves with different concentrations of gallic acid at 760 nm (UV-2600, Shimadzu Co., Kyoto, Japan). TFC determination was performed by adding 5% NaNO_2_ (0.15 mL) and 10% AlCl_3_ (0.15 mL) solutions to the extract (1 mL), and then adding 1 M NaOH (1 mL) for color development after avoiding light for 5 min. TFC in the sample was quantified by making standard curves with different concentrations of quercetin at 506 nm (UV-2600, Shimadzu Co., Tokyo, Japan). TPCs and TFCs were calculated as equivalents of gallic acid and rutin per kilogram of dry matter, expressed as g GAE/kg DM and g QE/kg DM, respectively.

Ascorbic acid (AsA) determination was performed by HPLC (Kim et al., 2021). First, the pulp sample (2 g) was mixed and ground with 10% phosphoric acid. Ultrasonic extraction (10 °C, 500 W, 30 min) was then conducted with water as the medium, followed by filtration of the extract through a 0.45-μm membrane filter. AsA was separated using an XBridge C18 column (5 μm, 4.6×250 mm; Waters Co., Milford, MA, USA), and an Alliance HPLC system equipped with a UV detector (Waters Co., Milford, MA, USA). The mobile phase consisted of 0.05 M potassium phosphate (pH 2.5) and methanol (90:10, v/v) at a flow rate of 1.0 mL/min. AsA was quantified at 254 nm using a standard curve of 0.1–20 mg/mL, and calculated as ascorbic acid per kilogram of dry matter (g/kg DM).

#### 2.3.2 Detection of individual phenolic components by liquid chromatography–electrospray ionization tandem mass spectrometry (LC-ESI-MS/MS)

Individual phenolic components were qualitatively determined using an LC-ESI-MS/MS system (Agilent 1100-API4000; Agilent Co., CA, USA) and quantitatively determined using standard products. The Agilent Poroshell 120 EC-C18 column (2.7 μm, 3 mm × 50 mm) was kept at 35 °C. The mobile phase consisted of 0.5% formic acid in water (A) and acetonitrile (B) with a flow rate of 0.6 mL/min. The following gradient elution procedure was used: 5% B at 0–2 min, 5%–25% B at 2–8 min, 25%–60% B at 8–12 min, 60%–100% B at 12–16 min, and 100%–5% B at 16–20 min. The ESI source of the mass spectrometer operated in negative ion mode, the spray voltage of the instrument was 5.5 kv; desolvation temperature was 500 °C; desolvation gas (N2) was 1000 L/h; scanning quality range: 100–1000 *m*/*z*. All compounds were identified by comparing dynamic multiple reaction monitoring information, including retention time, MS, and fragment ions.

#### 2.3.3 Antioxidant capacity

The antioxidant activity of jujube fruit was detected using DPPH, ABTS, and FRAP Kits (Beyotime, Shanghai, China). The standard curve was prepared with Trolox, and the antioxidant activity was expressed as Trolox equivalents per kilogram of jujube fruit pulp (dry weight) (M Trolox/kg DW).

### 2.4 Analysis of volatile components by headspace solid-phase microextraction–gas chromatography–mass spectrometry (HS-SPME-GC–MS)

#### 2.4.1 HS-SPME extraction of volatile components

To an extraction bottle containing the sample (4 g) was added ddH_2_O (5 mL) and 4-methyl-2-pentanol internal standard solution (50 μL, 0.305 mg/mL). The sample vials were preheated using the solid-phase microextraction unit at 60 °C for 20 min, and then SPME fibers (50/30 μm PDMS/CAR/DVB; Supelco Co., PA, USA) were inserted into the headspace of the sample and extracted for 30 min.

#### 2.4.2 Quantification of volatile components by GC–MS

VOCs were qualitatively and quantitatively determined using a 7890A-5975C GC–MS system equipped with a DB-WAX column (0.25 μm, 30.0 m × 250 μm; Agilent Co., CA, USA). The initial column temperature was 40 °C (maintained for 3 min), followed by an increase to 100 °C at a rate of 5 °C/min, and then 230 °C at 10 °C/min (maintained for 15 min). The gasification chamber and transmission line temperatures were 250 and 230 °C, the carrier gas was He with a flow rate of 1.0 mL/min. The mass spectrometer was set to electron ionization (EI) mode, with an ion source temperature of 230 °C, quadrupole temperature of 150 °C, and scanning quality range of 33–400 *m*/*z*. The volatile compounds were identified by mass spectra from NIST 17.0 database.

### 2.5 Fatty acids

The extraction and methylation of fatty acids in jujube were conducted according to the method of Song et al. (2019). Fatty acid methyl esters were quantified by an Agilent 7890A-5975C GC–MS system (Agilent Co., CA, USA) equipped with a HP-5MS column (60.0 m × 250 μm, 0.25 μm) column with reference to a fatty acid methyl ester mixed standard. The injection port temperature was 280 °C, the injection volume was 1.0 μL, the split ratio was 20:1, and the carrier gas was He at a flow rate of 1.5 mL/min. The initial column incubator temperature was 120 °C (maintained for 1 min), followed by an increase to 170 °C at a rate of 6 °C/min, 215 °C at 2.5 °C/min (maintained for 12 min), 230 °C at 4 °C/min (maintained for 10 min), and finally to 280 °C at 10 °C/min (maintained for 15 min). The mass spectrometer was set to electron ionization (EI) mode with an ion source temperature of 200 °C, quadrupole temperature of 150 °C, and scanning quality range of 40–550 *m*/*z*.

### 2.6 Amino acids

Amino acids were determined using an HPLC pre-column derivatization method. To a 10-mL centrifuge tube containing the sample (1 g) was added 0.01 M hydrochloric acid (5 mL), followed by heating in a water bath at 100 °C for 30 min. The extracted sample was centrifuged at 10,000 rpm for 10 min to obtain the free amino acid extract. Pre-column derivatization of the free amino acids was performed using the Agilent automatic online derivatization mode. Before injection, the amino acids were derivatized with *o*-phthalaldehyde (OPA) and fluorene methoxycarbonyl chloride (FMOC), followed by detected using the column. A ZORBAX Eclipse AAA amino analysis column (3.5 μm, 4.6×75 mm) and Agilent 1100 HPLC system with a variable wavelength detector (VWD) were used (Agilent Co., CA, USA). The mobile phase was composed of 0.04 M sodium dihydrogen phosphate (pH 7.8; A) and acetonitrile–methanol–water (45:45:10, v/v/v; B) at a flow rate of 1 mL/min. The following gradient elution procedure was used: 0% B at 0–23 min, 0%–57% B at 23–27 min, 57%–100% B at 27–40 min, and 100%–0% B at 40–45 min. The VWD detector detected ultraviolet light at 338 nm (0–19 min) and 266 nm (19–25 min).

### 2.7 Soluble monosaccharides

The sample (5 g) was mixed with deionized water (10 mL), ground and homogenized, and centrifuged with ultrasonication (10 °C, 500 W, 30 min) at 10,000 rpm for 10 min, and the supernatant was then collected. This extraction process was repeated twice, and the extract was set to a volume of 50 mL. Glucose, fructose, and sucrose were determined using a UPLC Q Exactive Plus mass spectrometer (Thermo Fisher Scientific Inc., MA, USA) equipped with a chromatographic column (Accucore 150 Amide HILIC; 2.6 μm, 150×2.1 mm). The mobile phase comprised acetonitrile–water (84:16, v/v; A) and 10 mM ammonium formate (B), with isocratic elution performed at a flow rate of 0.8 mL/min. The column temperature was 70 °C, the injector temperature was 4 °C, and the injection volume was 10 μL. DD MS2 full mass mode was used to scan and monitor substances with molecular weights of 150, 180, and 342. The ESI source (ESI-MS) ionized glucose, fructose, and sucrose in negative ion mode.

Rhamnose, mannose, xylose, galactose, and other reducing sugars were analyzed by 1-phenyl-3-methyl-5-pyrazolone (PMP)-derivatized HPLC according to (Liu et al., 2021a).

### 2.8 Statistical data

All determinations were conducted in triplicate. Principal component analysis and cluster analysis using Omicsshare 6.4.5 online analysis (Genedenovo Co.ltd, Guangzhou, China). The results are expressed as means ± standard deviation (SD). One-way analysis of variance was used to analyze all data. Differences between means were evaluated by Duncan’s multiple range tests using SPSS 20.0 (SPSS Inc., Chicago, IL, USA) with *P* < 0.05 indicating statistical significance.

## 3 Results and discussion

### 3.1 Phenolics and flavonoids

#### 3.1.1 TPC and TFC

**Fig. 1:**
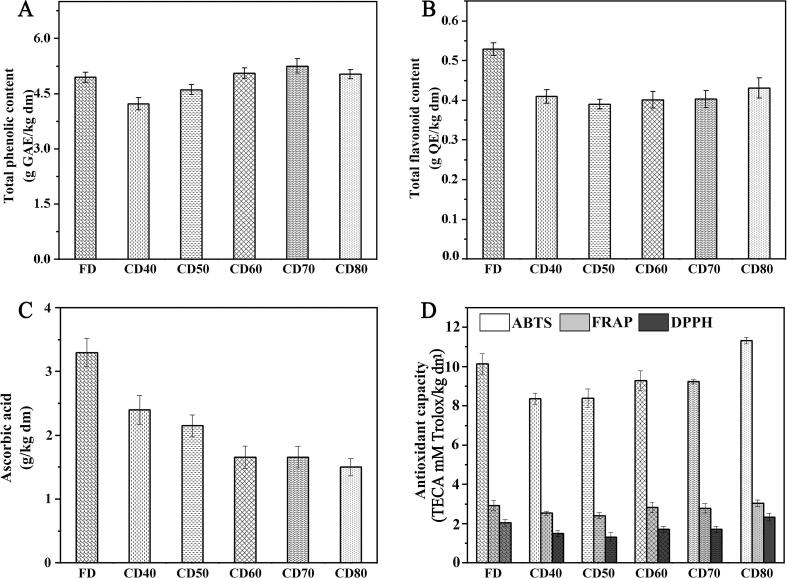
Effects of different drying methods on bioactive components and antioxidant capacity of jujube fruit. (A) Influence of convection drying and freeze drying on total phenolic components. (B) Influence of convection drying and freeze drying on total flavonoids components. (C) Influence of convection drying and freeze drying on ascorbic acid. (D) Effects of different drying methods on antioxidant capacity of jujube fruit.

Generally, bioactive components are protected during FD under the protection of low temperatures and pressures, while bioactive substances are susceptible to degradation by the combined effects of air and heat during CD. The TPC (Fig. 1a) was in the range of 4.21–5.24 g/kg, there was no significant difference in FD, CD60, CD70 and CD80 samples (*P* > 0.05). However, the TPC of CD40 and CD50 decreased significantly (*P* < 0.05) by 14.8% and 8.9% respectively compared with FD. The TFC range was 0.39–0.53 g/kg (Fig. 1b), with FD giving the highest value, followed by CD80. In the CD process, the plant cell structure is gradually broken, causing phenolics to be released from the cell matrix (Teribia et al., 2021). Phenolic release drives substrate exposure to phenol oxidase and peroxidase, which provokes a strong enzymatic reaction, leading to the extensive phenolic depletion of samples CD40 and CD50 (Chumroenphat et al., 2021). In contrast, no significant difference was observed between the TPC and TFC values of samples CD60, CD70, CD80, and FD (*P* > 0.05). The enzymatic effect of the reaction system might be inhibited by the high temperature, which protects phenols in the system from being consumed by enzymatic. Furthermore, jujube has not only free phenols that can be directly extracted, but also bound phenols connected with oligosaccharides or polysaccharides (such as lignin and tannin) through ester bonds (Zia & Alibas, 2021). With the increase in temperature, the ester bonds break from thermal shock, causing bound phenol to be transformed into free phenol, resulting in increased extractable phenols. At this time, phenolics might be involved in a dynamic equilibrium of self-pyrolysis and the conversion of bound phenolics to free phenolics, resulting in no significant difference. The same results were verified by Alkaltham et al. (2021), who reported a nearly four-fold increase in the TPC of convection-dried avocados relative to fresh samples. The authors attributed this trend to cell wall destruction during drying, which increased the solvent migration of phenol. These results show that CD60, CD70, and CD80 had a similar retention ability for jujube bioactive components to FD.

#### 3.1.2 Phenolic components

To further clarify changes in the phenolic compounds in jujube fruit, the composition and concentration of phenolics in the extract were studied by LC-ESI-MS/MS. Table 1 shows 16 phenolic substances identified in dried jujube. Qualitative information regarding the mass spectrometry of each substance is provided in the Table S1 (Supplementary Material). Rutin, *p*-hydroxybenzoic acid, and chlorogenic acid were the most abundant phenolic compounds in jujube. Compared with FD treatment, the rutin, epicatechin, and quercetin contents increased significantly after CD treatment (*P* < 0.05). It should be note that the rutin content of CD40–80 was 1.38, 1.43, 1.91, 2.12, and 2.01-fold higher than that of FD, respectively, and quercetin was also increased by 19.6–132.1%. Bao et al. (2021) also observed this phenomenon when drying fresh jujube fruit. Regarding the increase two phenolic compounds, thermal action may have triggered covalent cleavage of the corresponding bound phenolics into free states. At the same time, the thermal damage to the cell wall promotes the outward diffusion of these phenolic compounds from the cellular matrix. Furthermore, phenolic acids with *ortho-*hydroxy groups (caffeic acid and chlorogenic acid) have stronger ability to bind hydrogen ions, protecting quercetin and rutin from oxidation during drying, which further promotes their accumulation (Ma et al., 2021). Therefore, the quercetin and rutin contents of CD were higher than FD. The content of rutin and quercetin reached the highest value at CD70, however, they were significantly lower than CD70 at CD80, which may indicate that temperatures above 80 ℃ will cause rapid degradation and break the balance of phenolic compounds. Similar results have also been reported in the drying of tomatoes, litchi, and apples (Tan et al., 2021). On the contrary, in previous studies, the content of phenolic compounds such as rutin and quercetin decreased after dehydration of fruits such as rosehips and grapes (Goztepe et al., 2022, Qin et al., 2020). From this, we speculate that the difference in the composition of bound phenols is the reason for the inconsistent change trend of phenolics in different fruits after drying. Regarding the caffeic acid, chlorogenic acid, and *p*-hydroxybenzoic acid contents decreased significantly after CD treatment (*P* < 0.05), we speculated that these compounds might be involved in the Maillard reaction caused by thermal action. Previous studies have reported that these phenolic acids can specifically bind to the intermediate Maillard reaction products and delay the Maillard process (Chen et al., 2016, Li et al., 2019).

**Table 1:** Concentrations of phenolic compounds in jujube fruit from different drying methods.

Parameters mg/kg dm		Drying conditions					
		FD (Control)	CD40	CD50	CD60	CD70	CD80
1	Caffeic acid	30.84 ± 3.96a	24.35 ± 0.86b	20.18 ± 2.86c	13.48 ± 1.15e	14.245 ± 0.85de	16.66 ± 1.22d
2	Ferulic acid	2.75 ± 0.53b	2.03 ± 0.17c	3.45 ± 0.56a	2.25 ± 1.021	2.76 ± 0.55b	1.82 ± 0.11c
3	Phloroglucinol	6.38 ± 0.84bc	3.99 ± 0.43d	6.19 ± 1.13c	7.17 ± 0.61b	8.25 ± 1.15b	10.05 ± 0.58a
4	Cinnamic acid	12.64 ± 1.21a	11.65 ± 1.55a	11.46 ± 1.31a	11.63 ± 2.39a	11.37 ± 2.01a	11.86 ± 1.25a
5	Chlorogenic acid	67.25 ± 4.41a	48.85 ± 5.19b	44.33 ± 4.88bc	38.85 ± 2.94c	40.95 ± 0.63c	27.88 ± 4.04d
6	Hydroxybenzoic acid	37.95 ± 0.4.59a	35.3 ± 5.18a	31.45 ± 3.64a	33.4 5 ± 5.94a	31.45 ± 2.01a	24.3 ± 3.44b
7	*p*-Coumaric acid	5.33 ± 0.09b	4.23 ± 0.23d	4.7 ± 0.15c	8.29 ± 0.36a	5.35 ± 0.54b	5.17 ± 0.34bc
8	Vanillic acid	0.31 ± 0.13b	0.33 ± 0.12b	0.35 ± 0.09b	0.35 ± 0.01b	0.42 ± 0.11a	0.44 ± 0.03a
9	Gallic acid	14.31 ± 1.21a	10.22 ± 1.66c	10.21 ± 2.32c	10.85 ± 1.14c	11.34 ± 2.33bc	12.32 ± 1.06b
10	Catechin	4.35 ± 0.58c	5.99 ± 0.69ab	5.1 ± 0.56b	4.83 ± 0.47bc	5.48 ± 0.71b	6.22 ± 0.57a
11	Protocatechuic acid	18.42 ± 2.81c	26.1 ± 2.95b	28.4 ± 2.27b	31.3 ± 1.71ab	32.35 ± 2.91a	32.55 ± 2.11a
12	Epicatechin	7.75 ± 0.11a	6.21 ± 0.27c	6.33 ± 0.74b	7.44 ± 1.35ab	7.55 ± 0.76ab	8.32 ± 1.79a
13	Rutin	82.81 ± 8.33d	114.75 ± 6.62c	118.85 ± 13.41c	158.12 ± 10.34b	176.83 ± 8.43a	166.95 ± 14.33 ab
14	Quercetin	9.73 ± 0.84c	11.64 ± 1.85bc	19.31 ± 1.75a	20.08 ± 0.97a	20.15 ± 3.69a	22.58 ± 3.18a
15	Procyanidin B1	–	0.70 ± 0.08a	0.33 ± 0.03c	0.77 ± 0.11a	0.615 ± 0.15ab	0.55 ± 0.13b
16	Procyanidin B2	10.69 ± 1.24ab	12.21 ± 2.42a	8.88 ± 1.17b	12.38 ± 2.33a	8.18 ± 1.13c	10.43 ± 2.35ab

Data are shown as means ± standard deviation (n = 3). Different letters for the same component represent statistically significant differences (*P* < 0.05). ND, not detected.

### 3.2 Ascorbic acid (AsA)

AsA is a strongly reducing substance with a unique dienol structure that is potentially beneficial to humans, and is an important secondary metabolite in fruit growth. According to previously reported results, the AsA concentration in jujube fruit was 4–7 g/kg (Bao et al., 2022). In this study, the initial AsA concentration in jujube fruit was only 3.73 g/kg (Fig. 1c), which might be due to AsA being consumed during the long-term dry maturation process. The AsA concentration in FD was 3.29 g/kg, which was the highest among all dried samples. With an increasing CD temperature, the AsA concentration decreased significantly (*P* < 0.05), by 27.4%–54.7% compared with FD. This might be attributed to the influence of light and heat on the stability of AsA, resulting in the major loss of AsA during processing (Kim et al., 2021). As FD is conducted under closed low-temperature vacuum conditions, it effectively inhibits AsA degradation. Compared with the CD groups continuously exposed to hot air, FD remained the most efficient processing method for AsA retention.

### 3.3 Antioxidant capacity

ABTS and DPPH assays are antioxidant capacity evaluation methods that reflect the ability to scavenge ABTS^+^ and DPPH^+^ free radicals (Fig. 1D). The DPPH and ABTS scavenging ability ranges of CD60, CD70, CD80, and FD were 1.70–2.05 and 9.72–10.14 mM TE/g, respectively, with no statistically significant difference observed (*P* > 0.05). The antioxidant capacities of CD40 and CD50 were significantly lower than other samples (*P* < 0.05). The antioxidant capacity is affected by many factors, such as the composition and structure of active ingredients. Correlation analysis showed that ABTS was positively correlated with TPC (0.917) and DPPH was positively correlated with TFC (0.845), while AsA was not significantly correlated with antioxidant capacity. Xie et al. (2017) found a similar correlation in jujube fruit. Considering the characteristics of bioactive components, we proposed the presence of a synergistic effect between phenolics, which made a greater contribution to the antioxidant ability than AsA. Meanwhile, regarding single phenolic compounds, phloroglucinol, cinnamic acid, gallic acid, and epicatechin were significantly positively correlated with ABTS and DPPH, and might be the main providers of the antioxidant capacity in these samples.

The ferric reducing ability of plasma (FRAP) is an index for evaluating the reducing power of samples by determining the ability of samples to reduce Fe^3+^ to Fe^2+^. The results were similar to the previous free radical scavenging experiments, except for a significant increase in FRAP for CD80. Based on the determination principle of FRAP, we speculated that the Maillard reaction produces strong reducing components, which significantly improves the FRAP value (Patrignani et al., 2019).

### 3.4 Characterization of aroma by HS-SPME-GC–MS

**Fig. 2:**
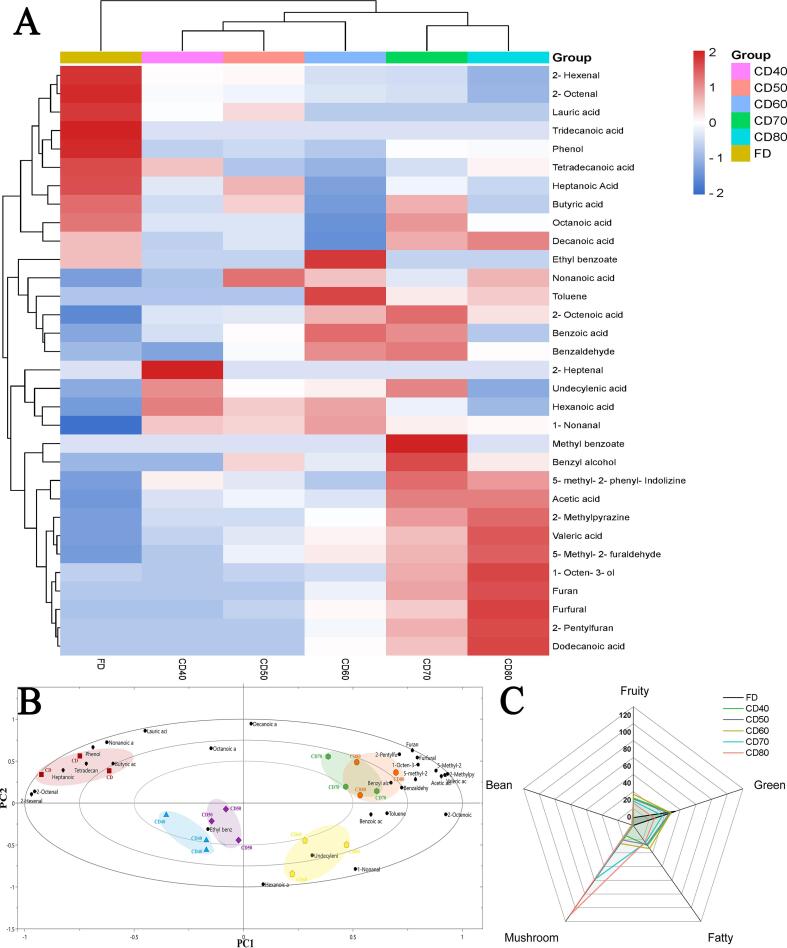
Effect of drying methods on volatile components of jujube. (A) Cluster heat maps of volatile components in convection dried and freeze dried samples. (B) Biplot for principal component analysis of convection dried and freeze dried samples. (C) Radar chart of sample aroma description.

A total of 31 possible VOCs were identified in jujube fruit (Fig. 2), with the specific statistical information for single VOCs included in Table S2. In this experiment, the VOCs included 7 aldehydes, 15 acids, 2 alcohols, 2 esters, and 6 heterocyclic compounds. Aldehydes and acids were the main components in the samples, with concentration ranges of 222.3–589.9 and 1520.8–1956.7 μg/kg, respectively, accounting for more than 80% of the total VOCs. However, this amount contrasted with the previous research of Song et al. (2020), who believed that the acid in jujube fruit was the most important VOC, accounting for more than 90% of the total concentration. The reason for the discrepancy in the data might be attributed to the different maturity of the jujube fruit. According to their concentrations, the main VOCs in jujube fruit were 2-hexenal, 2-octenal, benzaldehyde, acetic acid, caproic acid, heptanoic acid, and octanoic acid. These substances have been identified in jujube in previous studies (Chen et al., 2018, Liu et al., 2021b) , but a high concentration of 2-octenal has not been reported in previous studies.

In the dried samples, FD, CD40, CD50, CD60, CD70, and CD80, the number of VOCs identified was 17, 23, 24, 25, 25, and 23, respectively. To our knowledge, low temperature and vacuum are considered to be the most suitable drying methods for retaining fruit VOCs. Meanwhile, CD will cause the comprehensive chemical effect of heat, which will change the composition and concentration of fruit VOCs. Statistically, no significant difference was observed in the total VOC concentrations among samples FD, CD40, CD50, and CD60 (*P* > 0.05), while the total VOC concentrations of CD70 and CD80 were significantly higher than those of other samples (*P* < 0.05). Simply distinguishing the difference between samples using the VOC concentration and quantity is not sufficient. Therefore, we attempted to use principal component analysis (PCA) for VOCs analysis. PCA is an unsupervised clustering method that can reduce the dimension of data without the dataset. In the PCA model constructed, the PCs explained 83.5% (PC1, 54.2%; PC2, 29.3%) of the variance. The biplot of PCA is shown in Fig. 2B. FD was located in the second quadrant, CD40 and CD50 were located in the third quadrant, CD60 was located in the fourth quadrant, and CD70 and CD80 were located in the first quadrant. The loading showed that aldehydes and acids expressed fresh odor VOCs, which was the main feature of FD. The VOCs that appeared under thermal treatment were mostly distributed on the righthand side, and their appearance caused the separation of CD70 and CD80. Notably, CD60 was alone in the fourth quadrant, with characteristic VOCs of hexanoic acid, undecylenic acid, and 1-nonanal.

According to the VOC threshold, the odor activity value (OAV) of the dried samples was calculated (Table S3). Interestingly, although the concentration of acids in jujube fruit was the highest reported, the calculated OAV values of these substances were <1, and their contribution to the overall flavor could be largely ignored. After comparison, 6 VOCs with OAV greater than 1 were identified, namely, 2-hexenal, 1-nonanal, 3-octenal, benzaldehyde, 1-octen-3-ol, and 2-pentylfuran. The radar chart of aroma description after OAV superposition of these substances is shown in Fig. 2C. For jujube fruit, expression of the Fruit and Green aromas might be more acceptable to consumers (Wojdylo et al., 2016). Among the OAV >1 component, 2-hexenal, 1-nonanal, 3-octenal, and benzaldehyde had exactly this aroma type. However, with increasing CD temperature, 2-hexenal and 3-octene in jujube showed a decreasing trend, while the OAV decreased to 66.7%–100.0% and 64.9%–93.3% correspondingly. Compared with FD samples, 2-hexenal disappeared even in CD80. In contrast, 1-nonanal and benzaldehyde, which present Fruit, Green and Sweet aroma, accumulate in CD. This might mean that 1-nonanal and benzaldehyde replace 2-hexenal and 3-octenal, and express Fruit and Green aromas in the dried product. 2-pentylfuran, which appears when the drying temperature is higher than 60℃, is described as having the fragrance of beans, green and fruits, and is a typical Maillard reaction product. Furthermore, the OAV of 1-octen-3-ol, with an unpleasant oily aroma (Weng et al., 2021), was significantly increased when the CD temperature was greater than 70 °C, at almost 4 and 6 times that of FD. Therefore, we suppose that a drying temperature higher than 70 °C is unfavorable to the aroma of dried jujube. In general, we believe that the fruit and green aroma expressions of CD40, CD50, CD60, and FD were almost consistent. Considering that substances contributing to the aroma were all fatty acids or carbamide reaction products, the potential precursors were measured to further understand changes of VOCs.

### 3.5 Fatty acids

Fatty acids are important precursors of VOC. A total of 8 saturated fatty acids (SFAs) and 11 unsaturated fatty acids (USFAs) were identified in jujube fruit. These results (Table 2) were consistent with those described by Zhang et al. (2021). The saturated fatty acids (SFA) of jujube are mainly composed of two types, C18:0 and C16:0, accounting for about 90% of the SFAs. The representative USFAs were C18:1n9, C14:1n5, and C16:1n7, which accounted for more than 50% of USFA in jujube fruit. After CD treatment, almost all fatty acids were degraded, with C17:0, C18:1n7, C20:1n9m and C22:1n9 even disappearing in the CD samples. This might be attributed to the combined effects of heat and air intensifying α-oxidation, β-oxidation, and fatty acid oxidase pathways that degrade fatty acids into alcohols, aldehydes, ketones, acids, and esters (Fu et al., 2021). These products were also found in GC–MS, such as 1-nonanal with a Fruity and Green odor in CD (24.2–33.8 μg/kg), which was confirmed to be the product of C18:1n9 oxidative cleavage (Cao et al., 2020), as shown in Fig. S1. Benzaldehyde, with Sweet and Fruity odors, and benzoic acid with a Slight Sweet odor increased significantly with drying temperature (*P* < 0.05). According to Li et al. (2021), benzaldehyde might be the degradation product of C18:2n6. Furthermore, 1-octen-3-ol, which expresses a Mushroom odor, was increased in CD70 and CD80. Liu et al. (2020a) considered 1-octen-3-ol to be the degradation product of the secondary hydroperoxide thermochemical reaction of fatty acids. Notably, the USFA content decreased significantly in CD40 and CD50, by 36.1% and 56.6%, respectively, compared with FD, which might be due to the participation and acceleration of lipoxygenase (LOX) in the oxidation process. LOX mainly consumes USFAs and oxygen to generate C6 and C9 aldehydes (Luo et al., 2021), but only hexanoic and nonanoic acids were found in the C6 and C9 structural VOCs. We speculated that the long drying periods of CD40 and CD50 might contribute to the further oxidation of aldehyde to acids. In general, USFAs are important precursors that affect the aroma of jujube fruit during drying, and will be transformed into VOCs under the influence of CD temperature. However, these transformations have dual effects. When the drying parameters are unreasonable, some undesirable VOCs will be produced, such as 1-octen-3-ol. Therefore, we believe that CD above 70 °C promotes the excessive degradation of jujube fatty acids, which might be unfavorable for the quality of dried products.

**Table 2:** Fatty acids in jujube fruit from different drying methods.

	Compound mg/kg dm	Fatty acid	Drying conditions					
			FD (control)	CD40	CD50	CD60	CD70	CD80
1	Hexanoic acid	C6:0	3.98 ± 0.48c	4.21 ± 0.92c	3.83 ± 0.77c	6.73 ± 0.31a	5.33 ± 0.82b	6.58 ± 0.43a
2	Decanoic acid	C10:0	4.36 ± 0.22b	2.25 ± 0.46d	2.42 ± 0.21d	5.67 ± 0.33a	2.28 ± 0.21d	3.78 ± 0.17c
3	Lauric acid	C12:0	8.45 ± 1.21bc	5.35 ± 0.78d	6.45 ± 0.75c	10.06 ± 1.41a	7.36 ± 1.32c	10.96 ± 1.63a
4	9-Tetradecenoic acid	C14:1 n5	35.44 ± 3.26a	12.85 ± 1.32d	15.26 ± 2.21bc	18.46 ± 2.56c	16.32 ± 2.36c	23.56 ± 3.36b
5	Myristic acid	C14:0	3.04 ± 0.36d	6.77 ± 2.35ab	5.32 ± 0.68b	4.56 ± 0.21c	3.94 ± 0.36c	7.09 ± 1.21a
6	7-Hexadecenoic acid	C16:1 n9	21.18 ± 2.65b	8.69 ± 0.98c	13.31 ± 0.44b	22.28 ± 2.85a	18.45 ± 3.32a	22.97 ± 3.66a
7	9-Hexadecenoic acid	C16:1 n7	27.53 ± 4.31b	12.48 ± 3.26b	18.31 ± 6.33b	30.72 ± 3.58a	28.12 ± 5.21a	32.69 ± 4.69a
8	Palmitic acid	C16:0	208.65 ± 23.69a	137.57 ± 11.31d	157.92 ± 11.32c	172.08 ± 23.33bc	177.47 ± 10.32b	174.57 ± 12.36bc
9	7,10-Hexadecadienoic acid	C16:2 n6	14.71 ± 1.71b	ND	ND	13.66 ± 5.62a	12.36 ± 1.69a	12.33 ± 2.69a
10	Heptadecanoic acid	C17:0	1.38 ± 0.76a	ND	ND	ND	ND	ND
11	Linoleic acid	C18:2 n6	17.82 ± 3.69	9.32 ± 3.48c	8.78 ± 2.49c	14.44 ± 4.58ab	12.62 ± 0.96b	12.44 ± 1.69b
12	Oleic acid	C18:1 n9c	45.08 ± 7.31a	26.58 ± 5.63c	26.52 ± 5.69c	37.33 ± 7.21ab	35.42 ± 2.32b	33.08 ± 3.99b
13	9-Octadecenoic acid	C18:1 n9t	10.02 ± 3.58a	ND	ND	10.75 ± 1.36a	8.08 ± 2.33a	12.01 ± 1.66a
15	11-Octadecenoic acid	C18:1 n7	3.86 ± 0.62a	ND	ND	ND	ND	ND
16	Stearic acid	C18:0	109.40 ± 12.69a	76.20 ± 6.36c	81.34 ± 7.56bc	87.37 ± 8.99b	92.75 ± 6.99b	87.45 ± 9.21b
17	11-Eicosenoic acid	C20:1 n9	7.93 ± 1.99a	ND	ND	ND	ND	ND
18	Arachidic acid	C20:0	2.03 ± 1.21a	ND	ND	ND	ND	ND
19	Erucic acid	C22:1 n9	15.72 ± 7.31a	ND	ND	ND	ND	ND
	Saturated fatty acid (SFA)		341.29 ± 41.44a	232.35 ± 22.18d	257.28 ± 21.29c	286.47 ± 34.58b	289.13 ± 20.02b	290.43 ± 25.01b
	Unsaturated fatty acid (USFA)		189.29 ± 32.17a	69.92 ± 14.66c	82.18 ± 13.14c	146.87 ± 27.76b	131.37 ± 15.87b	149.06 ± 21.74b
	Total fatty acid (TFA)		540.58 ± 73.61a	302.27 ± 36.84c	339.46 ± 38.43c	433.346 ± 62.34b	420.50 ± 35.89b	439.49 ± 46.75b

Data are shown as means ± standard deviation (n = 3). Different letters for the same component represent statistically significant differences (*P* < 0.05). ND, not detected.

### 3.6 Amino acids

Amino acids are not only the basic units of proteins, but also precursors of taste and aroma. Table 3 shows the effects of different drying methods on the amino acid composition of jujube fruit. A total of 22 free amino acids (FAA) were identified in FD samples. The three most abundant FAAs were Pro (9946.3 mg/kg), Arg (392.9 mg/kg), and Asp (379.4 mg / kg), which was consistent with the previous report of Song et al. (2019). After CD treatment, almost all FAAs were degraded and the total amino acids in CD40, CD50, CD60, CD70, and CD80 were 84.2%, 81.6%, 68.8%, 62.6%, and 40.0% of those in FD with increasing temperature, respectively. This trend suggests that an increase in drying temperature is detrimental to the retention of FAA content in jujube fruit. Notably, Asp is the only amino acid that increased in content during heating and drying, with a significant increase of 3.16%–23.2% compared with FD samples (P < 0.05). Asp is considered to be the most inactive amino acid in the Maillard reaction, and its content might be accumulated with protein cleavage (Yang et al., 2019). The pyrolysis of amino acids in fruit includes dehydrogenation, decarboxylation, amino transfer, and the Maillard reaction. However, only typical Maillard reaction products furfural (0–184.8 μg/kg), furan (0–13.5 μg/kg), 5-methyl-2-furanal (0–49.4 μg/kg), and 2-methylpyrazine (0–65.9 μg/kg) were detected by GC–MS, meaning that the majority of amino acids were degraded owing to their participation in the Maillard reaction. The correlation results also showed a significant negative correlation between most amino acids and Maillard reaction products, which might be the common intermediates of multiple Maillard reaction pathways (Liu et al., 2020b, Yu and Zhang, 2010, Zhang et al., 2019).

**Table 3:** Amino acids and sugars in jujube fruit from different drying methods.

Parameters	Drying conditions					
	FD (control)	CD40	CD50	CD60	CD70	CD80
Free amino acids (mg/kg dm)						
Asp	379.4 ± 29.3b	391.1 ± 42.9b	400.8 ± 69.7b	405.6 ± 47.9b	497 ± 21.3a	467.9 ± 43.4ab
Glu	304.8 ± 14.1a	245 ± 12.9b	223.7 ± 38.1bc	201.1 ± 20.7c	202.7 ± 39.5c	204 ± 17.1c
Ser	363.2 ± 31.9a	272.8 ± 21.3b	212.4 ± 22.7c	177.7 ± 19.1c	99.8 ± 18.5d	44 ± 7.4e
Gln	44.9 ± 2.9a	30.8 ± 7.7b	21.1 ± 2.2c	14 ± 1.3d	7.7 ± 0.9e	–
His	77.7 ± 5.7a	67.6 ± 3.7a	50 ± 6.8b	42.6 ± 9.6b	61.4 ± 3.8a	46.5 ± 3.6b
Gly	65.9 ± 6.1a	41.2 ± 4.5b	36.3 ± 2.1bc	31.0 ± 5.5c	25.2 ± 1.9c	19.2 ± 1.1d
Thr	210.4 ± 20.2a	173.8 ± 33.0b	116.9 ± 14.2c	86.4 ± 6.6d	45.7 ± 6.5e	25.3 ± 4.3f
Cit	60.5 ± 5.1a	44.2 ± 5.1b	22.6 ± 3.3c	13.2 ± 2.1d	6.5 ± 1.8e	ND
Arg	392.9 ± 26.9a	297.2 ± 16.7b	219.6 ± 40.4c	154.3 ± 12.5c	126.5 ± 13.3d	81.6 ± 6.1d
Ala	177.9 ± 16.8a	144.2 ± 19.4a	140.7 ± 11.6b	125.9 ± 18.9bc	93.4 ± 10.2c	62.6 ± 2.3d
Tyr	52.5 ± 1.1d	8 ± 1.8d	12.1 ± 1.2d	23.8 ± 2.6c	32.8 ± 2.1b	42.7 ± 4.6a
Cys	79.2 ± 5.6a	48.8 ± 4.2b	48.1 ± 5.89b	21.5 ± 7.2c	ND	ND
Val	174.9 ± 16.6a	142.6 ± 18.9b	103.8 ± 12.5c	85.3 ± 3.3c	63.8 ± 7.2d	43.1 ± 1.9e
Nva	299.3 ± 16.6a	172 ± 20.8b	155.2 ± 18.5b	101.8 ± 11.1c	61.4 ± 7.6d	30.8 ± 6.3e
Trp	107.3 ± 8.1a	81.5 ± 3.1b	73.5 ± 4.2c	60 ± 10.1c	54.4 ± 7.4d	49.2 ± 7.1d
Phe	65.4 ± 2.0a	47.3 ± 1.7b	34.7 ± 6.5c	26.7 ± 2.3c	17.5 ± 1.8d	12 ± 1.1e
Ile	57.1 ± 6.1a	37.8 ± 8.2b	30.5 ± 6.2bc	26.8 ± 5.5c	21.9 ± 2.5 cd	17.5 ± 2.8d
Leu	70.4 ± 5.8a	51.8 ± 5.8b	39.2 ± 6.9bc	32.2 ± 2.6c	23.2 ± 6.4d	13.2 ± 2.3e
Lys	40.3 ± 2.3a	26.9 ± 2.2b	20 ± 3.2c	11.2 ± 2.3d	4.1 ± 1.4e	ND
Hyp	276.6 ± 13.9a	221.9 ± 20.4b	193.1 ± 15.5bc	172.6 ± 24.2c	153.7 ± 6.9c	113.6 ± 15.3d
Sar	39.1 ± 2.2a	32.7 ± 3.4b	32.3 ± 2.6b	28.2 ± 8.4bc	21.1 ± 4.5c	11.2 ± 2.2d
Pro	9946.3 ± 803.4a	8610.4 ± 947.1a	8661.0 ± 637.3a	7306.9 ± 529.5b	6701.0 ± 549.5b	4030.5 ± 363.5c
Total	13286.0 ± 1042.7a	11189.6 ± 1204.8b	10847.6 ± 931.9b	9148.8 ± 753.3c	8320.8 ± 715.1c	5314.9 ± 492.3d

Sugars (g/kg dm)						
Glucose	219.1 ± 14.1b	232.7 ± 18.5b	289.5 ± 21.1a	263.5 ± 21.9ab	251.5 ± 23.2ab	262.1 ± 21.4ab
Sucrose	271.3 ± 17.2a	182.3 ± 21.3b	184.4 ± 17.9b	197.5 ± 14.7b	173.5 ± 17.7b	122.0 ± 9.7c
Fructose	136.1 ± 7.3c	204.2 ± 24.8b	225.7 ± 26.6b	236.7 ± 15.8b	254.3 ± 13.3ab	272.4 ± 13.5a
Rhamnose	1.7 ± 0.1a	0.6 ± 0.1c	1.1 ± 0.1b	1.2 ± 0.1b	1.1 ± 0.1b	0.7 ± 0.1c
Mannose	16.2 ± 1.1a	10.2 ± 1.3b	7.8 ± 0.3c	6.2 ± 0.6d	5.0 ± 0.8d	3.7 ± 0.3e
Xylose	2.8 ± 0.1a	1.0 ± 0.1b	ND	ND	ND	ND
Galactose	3.30 ± 0.2a	3.1 ± 0.1ab	3.1 ± 0.1ab	3 ± 0.1b	2.1 ± 0.1c	2.3 ± 0.1c
Total free sugar	650.4 ± 40.1a	634.1 ± 66.2a	741.6 ± 66.1a	708.1 ± 53.1a	687.5 ± 55.2a	663.2 ± 45.1a

Data are shown as means ± standard deviation (n = 3). Different letters for the same component represent statistically significant differences (*P* < 0.05). ND, not detected.

### 3.7 Soluble monosaccharides

Among macronutrients, more than 60% of mature jujube is composed of soluble sugar, which provides sufficient precursors for the Maillard reaction. Statistical data regarding soluble sugar in the dry samples are shown in Table 3. Consistent with the report of Pu et al. (2018), the most abundant sugar in jujube fruit was sucrose, followed by fructose and glucose, which together constituted more than 95% of soluble sugar. Statistical analysis showed that the sucrose, rhamnose, mannose, xylose, and galactose contents in CD samples decreased with increasing drying temperature. These pentoses and hexoses might be consumed as substrates in the Maillard reaction. Among them, xylose, as the most active monosaccharide in the Maillard reaction, was found only in samples FD and CD40. Glucose and fructose are supposed to be consumed in the Maillard reaction, which shows a tendency to increase with increasing temperature (*P* < 0.05). Combined with the change in sucrose, we speculated that high temperatures induced the hydrolysis mechanism of sucrose, which was decomposed into glucose and fructose (Sun et al., 2019), masking the consumption of glucose and fructose in the Maillard reaction. Interestingly, according to the latest report, if the sweetness of a molecule of sucrose is defined as 1, the sweetness of glucose and fructose are 1.12 and 0.94, respectively (Mao et al., 2019), which means that the hydrolysis of a sucrose molecule will produce approximately twice the original sweetness. Therefore, CD might make the sweetness of jujube more prominent. In the sensory evaluation of jujube fruit reported by Wojdylo et al. (2016), sweetness is directly related to quality. Overall, this study indicated that, although CD changes the soluble sugar composition of jujube, it does not change or even enhance the original sweetness. Meanwhile, soluble sugar might be used as a precursor to produce a series of Maillard reaction products with baking aroma, which plays a positive role in the overall flavor of jujube fruit.

## 4 Conclusion

The evaluation of bioactive and volatile components of jujube by CD and FD showed that inappropriate drying methods would reduce the quality of dried products. With the transformation of bound phenolics caused by increasing drying temperature, CD samples such as CD60, CD70, and CD80 can also reach the same levels of bioactive substances as FD. Regarding aroma, aldehydes, acids, alcohols, esters, furans, and pyrazines were the main volatile components of CD and FD dates. Among them, 2-hexenal, 1-nonanal, 3-octenal, benzaldehyde, 1-octen-3-ol, and 2-pentylfuran were the VOCs with OAV >1. Combined with the quantitative results regarding fatty acids, amino acids, and monosaccharides, the oxidation reaction and Maillard reaction of fatty acids were key to controlling the dried jujube aroma. The excessive oxidation of fatty acids caused by CD70 and CD80 produces an unwanted dry product flavor. Considering the retention of bioactive components and the acceptability of volatile components, convective drying at 60 °C was the most potentially effective drying method to replace FD. Considering the economic benefits of the process and the overall product quality, we plan to develop a multi-stage variable temperature drying process on this basis to continuously provide high-quality raw materials for market and processing enterprises.

## Data avail ability statement

The data that support the findings of this study are available on request from the corresponding author. The data are not publicly available due to privacy or ethical restrictions.

## CRediT authorship contribution statement

**Yuxing Liu:** Conceptualization, Investigation, Formal analysis, Writing – original draft. **Yaxuan Liao:** Investigation, Writing – review & editing. **Minrui Guo:** Formal analysis, Methodology, Writing – review & editing. **Weida Zhang:** Formal analysis, Methodology, Writing – review & editing. **Yueying Sang:** Methodology, Writing – review & editing. **Hai Wang:** Methodology, Writing – review & editing. **Shaobo Cheng:** Conceptualization, Resources, Supervision, Funding acquisition, Project administration, Writing – review & editing. **Guogang Chen:** Conceptualization, Resources, Supervision, Funding acquisition, Project administration, Writing – review & editing.

## Declaration of Competing Interest

The authors declare that they have no known competing financial interests or personal relationships that could have appeared to influence the work reported in this paper.
