# Supplementary material for: Comparative elucidation of bioactive and volatile components in dry mature jujube fruit (Ziziphus jujuba Mill.) subjected to different drying methods
Source: Food Chem X. 2022 Apr 18;14:100311. doi: 10.1016/j.fochx.2022.100311 (PMC9043666; doi:10.1016/j.fochx.2022.100311)
Supplement: Supplementary data 1 [file mmc1.doc]

| Table S1. Phenolic compounds identified in jujube fruit. | | | |
| --- | --- | --- | --- |
| **Compound** | **Ion mode** | **m/z** | **Product ions** |
| Caffeic acid | - | 180.2 | 135.1; 117 |
| Ferulic acid | - | 194.2 | 177; 134 |
| Phloroglucinol | - | 124.9 | 83; 57; 41 |
| Cinnamic acid | - | 147.1 | 103; 76.90 |
| Chlorogenic acid | - | 353 | 191 |
| *p*-Hydroxybenzoic acid | - | 136.9 | 93 |
| *p*-Coumaric acid | - | 162.8 | 92; 65 |
| Vanillic acid | - | 166.9 | 152.1; 108 |
| Gallic acid | - | 169 | 169 |
| Catechin | - | 289.1 | 245 |
| Protocatechuic acid | - | 152.9 | 123.1 |
| Epicatechin | - | 289 | 202; 109 |
| Rutin | - | 609 | 300; 270 |
| Quercetin | - | 300.8 | 178.9; 151 |
| Procyanidins B1 | - | 577 | 424; 407; 288 |
| Procyanidins B2 | - | 577.3 | 424; 406 ;288;124.9 |

| Table S2. Contents of volatile compounds in jujube fruit from different drying methods (μg/kg). | | | | | | | | | | | |
| --- | --- | --- | --- | --- | --- | --- | --- | --- | --- | --- | --- |
|  | Compounds ^a^ | Cas | RT ^b^ | LRI^c^ | Drying condition | | | | | | Aroma description^d^ |
|  |  |  |  |  | FD | CD40 | CD50 | CD60 | CD70 | CD80 |  |
|  | **Aldehydes** |  |  |  |  |  |  |  |  |  |  |
| 1 | 2-Hexenal | 505-57-7 | 13.79 | 1092 | 155.9±15.6 | 55.9±7.5 | 57.6±10.8 | 32.6±6.4 | 29.1±3.1 | - | Green, Fruity^1^ |
| 2 | 2-Heptenal | 2463-63-0 | 17.57 | 1251 | - | 4.0±0.9 | - | - | - | - | Herb, Green, Fatty^1^ |
| 3 | 1-Nonanal | 124-19-6 | 19.7 | 1306 | - | 29.4±8.5 | 27.9±3.6 | 33.8±3.4 | 25.3±4.9 | 24.2±3.2 | Green, Fruity, Fatty^5^ |
| 3 | 2-Octenal | 2363-89-5 | 20.63 | 1354 | 130.7±17.3 | 47.21±7.9 | 44.1±5.6 | 36.1±4.6 | 32.9±6.4 | 9.0±1.9 | Green^7^ |
| 4 | Furfural | 98-01-1 | 21.54 | 1418 | - | - | 17.4±2.82 | 66.57±13.13 | 95.9±12.7 | 184.9±13.9 | Sweet, Bread^1^ |
| 5 | Benzaldehyde | 100-52-7 | 22.8 | 1560 | 107.9±3.8 | 71.3±9.2 | 216.5±23.7 | 351.2±29.2 | 371.2±132.8 | 225.4±16.3 | Fruity^3^ |
| 6 | 5-Methyl-2-furaldehyde | 3857-25-8 | 23.7 | 1682 | - | 10.5±1.2 | 20.72±3.1 | 26.9±1.2 | 35.3±4.5 | 49.4±7.2 | NF |
|  | **Acids** |  |  |  |  |  |  |  |  |  |  |
| 7 | Acetic acid | 64-19-7 | 21.1 | - | 460.4±10.3 | 610.3±57.5 | 643.6±27.1 | 616.3±16.6 | 834.2±32.0 | 833.3±66.2 | Vinegar^3^ |
| 8 | Butyric acid | 107-92-6 | 24.41 | - | 43.2±12.0 | 20.8±9.1 | 32.1±10.8 | 10.1±3.2 | 35.6±19.1 | 18.4±8.1 | Cheesy^3^ |
| 9 | Valeric acid | 109-52-4 | 25.98 | 1518 | - | 34.2±14.1 | 46.5±24.3 | 66.3±27. | 86.9±8.2 | 131.3±12.4 | Rancid, sour^8^ |
| 10 | Hexanoic acid | [142-62-1](http://www.ichemistry.cn/chemistry/142-62-1.htm) | 27.33 | 1695 | 332.8±10.2 | 470.4±33.6 | 431.9±52.5 | 452.0±17.6 | 397.2±27.8 | 315.2±14.4 | Cheesy, Fatty^3^ |
| 11 | Heptanoic Acid | 111-14-8 | 28.56 | 1783 | 201.4±12.2 | 124.8±12.4 | 163.5±33.3 | 85.6±21.9 | 130.4±23.4 | 115.1±14.9 | Rancid, Greasy^3^ |
| 12 | Octanoic acid | 124-07-2 | 29.69 | 1897 | 221.2±24.1 | 152.3±11.2 | 152.4±18.7 | 101.7±11.6 | 258.2±122.1 | 165.5±10.3 | Rancid, Greasy^3^ |
| 13 | Undecylenic acid | 112-38-9 | 30.35 | 1792 | - | 10.7±9.7 | 5.8±2.1 | 6.3±0.4 | 11.1±5.6 | - | NF |
| 14 | Nonanoic acid | 112-05-0 | 30.77 | 1848 | 34.1±8.9 | 26.2±3.5 | 15.6±3.3 | 15.4±3.3 | 18.5±1.2 | 22.9±9.4 | Waxy, Cheesy^3^ |
| 15 | 2-Octenoic acid | 1871-67-6 | 31.02 | 1880 | - | 14.2±1.9 | 14.5±3.2 | 25.4±1.2 | 33.3±13.0 | 20.8±6.3 | NF |
| 16 | Decanoic acid | 334-48-5 | 31.94 | 888 | 77.8±12.4 | 51.5±4.2 | 57.8±15.9 | 32.6±3.7 | 80.9±10.4 | 88.3±3.7 | Fatty, Fruity^3^ |
| 17 | Lauric acid | 143-07-7 | 32.89 | 1077 | 87.9±6.7 | 23.3±3.4 | 36.8±12.0 | 21.9±5.6 | 37.1±4.8 | 69.4±3.8 | Fatty, Coconut^8^ |
| 18 | Benzoic acid | 65-85-0 | 34.44 | 975 | 32.9±4.3 | 44.2±4.3 | 50.4±2.7 | 69.9±1.8 | 104.3±10.2 | 39.3±6.9 | NF |
| 19 | Tridecanoic acid | 638-53-9 | 34.82 | 1073 | 25.6±1.7 | - | - | - | - | - | Waxy, Woody^8^ |
| 20 | Tetradecanoic acid | 57677-52-8 | 39.15 | 1256 | 36.1±6.8 | 21.4±1.94 | 3.1±1.3 | - | 8.07±0 | 15.3±3.8 | NF |
|  | **Alcohols** |  |  |  |  |  |  |  |  |  |  |
| 21 | 1-Octen-3-ol | 3391-86-4 | 20.98 | 1354 | 19.9±2.7 | 15.1±5.4 | 21.1±7.5 | 26.39±2.1 | 77.8±6.3 | 116.2±62.8 | Mushroom^7^ |
| 22 | Benzyl alcohol | 100-51-6 | 27.88 | 1663 | - | - | 6.0±2.6 | 3.2±0.2 | 11.6±1.8 | 5.1±0.1 | Flour^8^ |
|  | **Esters** |  |  |  |  |  |  |  |  |  |  |
| 23 | Methyl benzoate | 93-58-3 | 24.5 | 1527 | - | - | - | - | 7.2±2.1 | - | Herb^3^ |
| 24 | Ethyl benzoate | 93-89-0 | 25.17 | 1529 | 10.3±3.2 | - | - | 21.02±15.8 | - | - | Flour^3^ |
|  | **Others** |  |  |  |  |  |  |  |  |  |  |
| 25 | 5-Methyl-2-phenyl-indolizine | 36944-99-7 | 5.42 | - | - | 30.6±7.3 | 21.0±9.4 | 12.0±5.8 | 54.6±5.3 | 45.9±22.8 | NF |
| 26 | Toluene | 108-88-3 | 6.68 | 655 | - | - | - | 52.4±17.4 | 20.7±6.3 | 26.4±1.9 | Sweet^8^ |
| 27 | 2-Methylpyrazine | 109-08-0 | 7.31 | 672 | - | 20.6±3.3 | 21.0±9.4 | 32.0±5.8 | 54.6±5.3 | 65.9±13.8 | Fatty, Roast^2^ |
| 28 | 2-Pentylfuran | 3777-69-3 | 28.12 | 1668 | - | - | - | 3.3±1.2 | 7.3±1.5 | 12.2±4.7 | Bean, Fruity,Green^6^ |
| 29 | Furan | 110-00-9 | 28.76 | 1457 | - | - | - | 3.1±1.8 | 9.1±3.8 | 13.5±3.6 | NF |
| 30 | Phenol | 108-95-2 | 29.31 | 2088 | 23.7±40.1 | 6.6±0.5 | 7.2±0.7 | 5.7±1.5 | 10.6±3.2 | 10.4±2.4 | Rubber, Plastic ^3^ |
|  | **Total** |  |  |  | 2001.8±192.5 | 1895.5±218.5 | 2114.5±286.4 | 2209.7±223.43 | 2879.8±277.8 | 2623.3±314.8 |  |

Data are presented as means ± SD (n=3).

^a^ Volatile compounds detected were integrated with the GC-MS automatic deconvolution system and compared with the standard mass spectrum in the NIST 14 library. Each category of volatile compound is listed in order of retention time.

^b^ RT, retention time (min) of identified compounds on the DB-WAX capillary column.

^c^ LRI, linear retention index.

^d^ Aroma descriptions.

[1] Sun, XL. Wang, YJ. Li, HK. Zhou, J. Han, JJ. & Wei, CQ. (2021). Changes in the volatile profile, fatty acid composition and oxidative stability of flaxseed oil during heating at different temperatures. *LWT-Food Science and Technology*, 151, 112137. doi.org/ 10.1016/j.lwt.2021.112137

[2] Zhang, D. Ji, HW. Liu, SC & Gao, J. (2020). Similarity of aroma attributes in hot-air-dried shrimp (Penaeusvannamei) and its different parts using sensory analysis and GC-MS. *Food Research International*,137, 109507. doi.org10.1016/j.foodres.2020.109517

[3] Tan, FL, Wang, P, Zhan, P & Tian, HL. (2022). Characterization of key aroma compounds in flat peach juice based on gas chromatography-mass spectrometry-olfactometry (GC-MS-O), odor activity value (OAV), aroma recombination, and omission experiments. *Food Chemistry*, 366, 130604. doi.org/10.1016/j.foodchem.2021.130604

[4] Niu, YW. Deng, JM. Xiao, ZB & Zhu, JC. (2021). Characterization of the major aroma-active compounds in peach (*Prunus persica L.* Batsch) by gas chromatography-olfactometry, flame photometric detection and molecular sensory science approaches. *Food research international*, 147, 10457. doi.org/10.1016/j.foodres.2021.110457

[5] Ren, LY. Ma, J. Lv, Y. Tong, QG & Guo, HY. (2021). Characterization of key off-odor compounds in thermal duck egg gels by GC-olfactometry-MS, odor activity values, and aroma recombination. *LWT-Food Science and Technology*, 143, 111182. doi.org/10.1016/j.lwt.2021.111182

[6] Zhu, Y. Lv, HP. Shao, CY. Kang, SY. Zhang, Y. Guo, L. Dai, WD. Tan, JF. Peng, QH & Lin, Z. (2018).Identification of key odorants responsible for chestnut-like aroma quality of green teas. *Food research international*, 108, 74-82. doi.org/ 10.1016/j.foodres.2018.03.026

[7] Li, SH. Zhao, W. Liu, SY. Li, PL. Zhang, AX. Zhang, JL. Wang, YT. Liu, YY & Liu, JK. (2021). Characterization of nutritional properties and aroma compounds in different colored kernel varieties of foxtail millet (Setaria italica). *Journal of Cereal Science*, 100, 103248. doi.org/10.1016/j.jcs.2021.103248

[8] Aroma description from [http://www.perflavory.com](http://www.perflavory.com/)

| Table S3. Contents of volatile compounds in jujube fruit from different drying methods (μg/kg). | | | | | | | | | | |  | | |  | | | |
| --- | --- | --- | --- | --- | --- | --- | --- | --- | --- | --- | --- | --- | --- | --- | --- | --- | --- |
|  | Compounds^a^  ug/kg | Drying condition | | | | | | OT ^a^ | OVA ^b^ | | | | | | | | Aroma description |
|  |  | FD | CD40 | CD50 | CD60 | CD70 | CD80 | ug/kg | FD | CD40 | | CD50 | CD60 | | CD70 | CD80 |  |
|  |  |  |  |  |  |  |  |  |  |  | |  |  | |  |  |  |
| 1 | 2-Hexenal | 155.91 | 55.93 | 57.6 | 32.6 | 29.2 | ND | 17 | 9.2 | 3.3 | | 3.4 | 1.9 | | 1.7 | 0 | Green, Fruity |
| 2 | 1-Nonanal | ND | 29.35 | 27.89 | 23.8 | 25.3 | 24.2 | 1 | 0 | 29.4 | | 27.9 | 33.8 | | 25.3 | 24.2 | Green, Fruity, Fatty |
| 3 | 2-Octenal | 130.7 | 47.21 | 44.1 | 36.1 | 32.9 | 9.03 | 3 | 43.6 | 15.7 | | 14.7 | 12.0 | | 11.0 | 3.0 | Green |
| 4 | Benzaldehyde | 107.96 | 71.31 | 216.5 | 351.2 | 371.3 | 225.37 | 350 | 0 | 0 | | 0 | 1.0 | | 1.1 | 0 | Fruity |
| 5 | 1-Octen-3-ol | 19.9 | 15.01 | 21.36 | 26.3 | 77.84 | 116.72 | 1 | 19.9 | 15.1 | | 21.4 | 26.4 | | 77.8 | 116.7 | Mushroom |
| 6 | 2-Pentylfuran | ND | ND | ND | 3.3 | 4.32 | 12.21 | 5.8 | 0 | 0 | | 0 | 0 | | 1.3 | 2.1 | Bean, Fruity, Green |

^a^ Odor threshold.

[1] Pang, XL. Guo, XF. Qin, ZH. Yao, YB. Hu, XS & Wu, JH. (2012). Identification of Aroma-Active Compounds in Jiashi Muskmelon Juice by GC-O-MS and OAV Calculation. *Journal of Agricultural and Food Chemistry*, 60(17), 4179-4185. doi.org/10.1021/jf300149m

[2] Forero, DP. Orrego, CE. Peterson, DG & Osorio, C. (2015). Chemical and sensory comparison of fresh and dried lulo (Solanum quitoense Lam.) fruit aroma. *Food Chemistry*, 169, 85-91. doi.org/10.1016/j.foodchem.2014.07.111

[3] Zhu, CH. Lu, Q. Zhou, XY. Li, JX. Yue, JQ. Wang, ZR & Pan, SY. (2020). Metabolic variations of organic acids, amino acids, fatty acids and aroma compounds in the pulp of different pummelo varieties. *LWT-Food Science and Technology*, 130, 109445. doi.org/10.1016/j.lwt.2020.109445

[4] Yang, YN. Zheng, FP. Yu, AN & Sun, BG. (2019). Changes of the free and bound volatile compounds in Rubus corchorifolius L. f. fruit during ripening. *Food Chemistry*, 287, 232-240. doi.org/10.1016/j.foodres.2020.109517

[5] Zhang, JG. Li, M. Zhang, HF & Pang, XL. (2022). Comparative investigation on aroma profiles of five different mint (Mentha) species using a combined sensory, spectroscopic and chemometric study. *Food Chemistry*, 371, 131104. doi.org/10.1016/j.foodchem.2021.131104

^b^ Odor active value

| 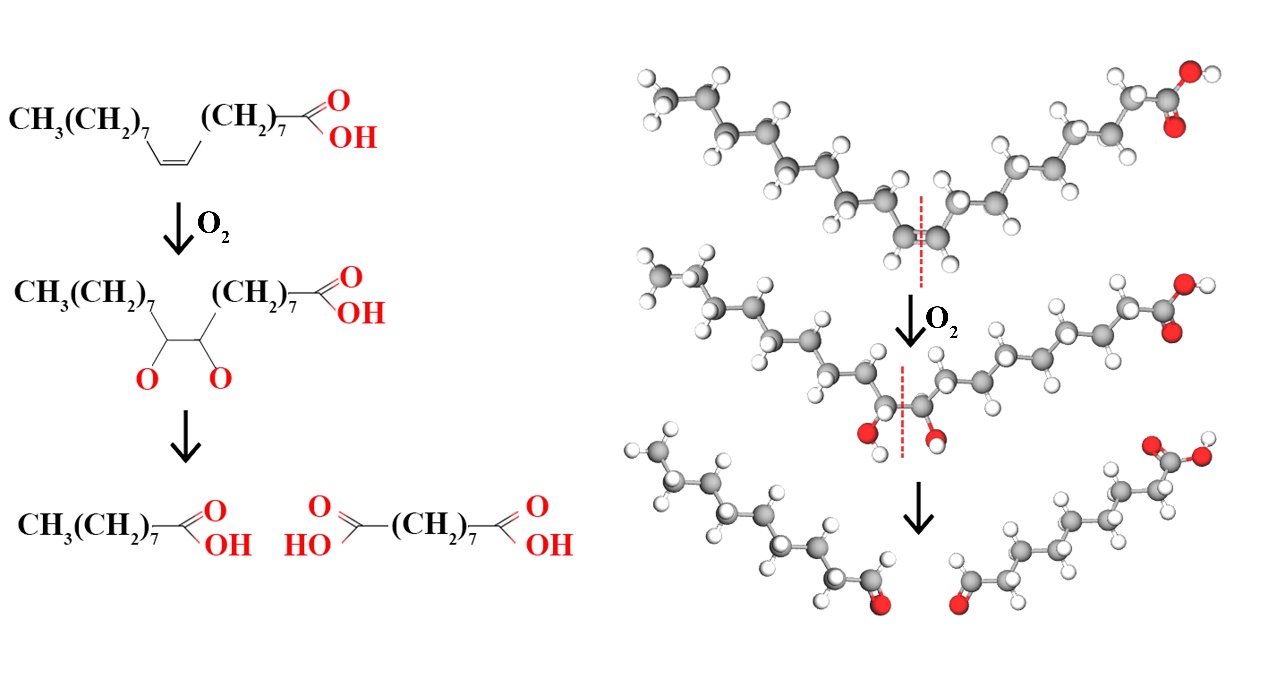 |
| --- |
| Fig. S1. Potential mechanism of 1-nonanal formation from c18:1n9 by oxidative bond cleavage. |
